# Supplementary material for: Alpha 1,3 N-Acetylgalactosaminyl Transferase (GTA) Impairs Invasion Potential of Trophoblast Cells in Preeclampsia
Source: Int J Mol Sci. 2024 Jul 2;25(13):7287. doi: 10.3390/ijms25137287 (PMC11242368; doi:10.3390/ijms25137287)
Supplement: Supplementary file 1 [file ijms-25-07287-s001.zip › ijms-2768179-supplementary.pdf]

Supplementary Table S1:

Clinical data from patients in the 1st trimester, 3rd trimester and preeclampsia groups

	**1st trimester**	**3rd trimester**	**Preeclampsia**
	**(n = 10)**	**(n = 10)**	**(n = 10)**
**Age**	25.05 ± 2.5	29.15 ± 3.0	28.05 ± 3.5
**Gestational weeks**	8W ± 2.0	35W ± 2.0	34W ± 2.0
**Systolic pressure (mm Hg)**	120 .00 ± 9.0	116 .00 ± 8.50	160.00 ± 9.55
**Diastolic pressure (mm Hg)**	75.0 ± 7.0	70.55 ± 6.30	95.25 ± 8.25
**Albuminuria (g/24 h)**	——	——	1.45 ± 0.69
